# Prolonged Impairment of Immunological Memory After Anti-CD20 Treatment in Pediatric Idiopathic Nephrotic Syndrome

**DOI:** 10.3389/fimmu.2019.01653

**Published:** 2019-07-16

**Authors:** Manuela Colucci, Rita Carsetti, Jessica Serafinelli, Salvatore Rocca, Laura Massella, Antonio Gargiulo, Anna Lo Russo, Claudia Capponi, Nicola Cotugno, Ottavia Porzio, Andrea Onetti Muda, Paolo Palma, Francesco Emma, Marina Vivarelli

**Affiliations:** ^1^Renal Diseases Research Unit, Genetics and Rare Diseases Research Division, Ospedale Pediatrico Bambino Gesù, IRCCS, Rome, Italy; ^2^Unit of Diagnostic Immunology, Unit of B-Cell Pathophysiology, Department of Laboratories, Immunology Research Area, Ospedale Pediatrico Bambino Gesù, IRCCS, Rome, Italy; ^3^Division of Nephrology, Department of Pediatric Subspecialties, Ospedale Pediatrico Bambino Gesù, IRCCS, Rome, Italy; ^4^Research Unit in Congenital and Perinatal Infections, Immune and Infectious Diseases Division, Academic Department of Pediatrics, Ospedale Pediatrico Bambino Gesù, IRCCS, Rome, Italy; ^5^Core Facilities, Ospedale Pediatrico Bambino Gesù, IRCCS, Rome, Italy; ^6^Medical Laboratory Unit, Ospedale Pediatrico Bambino Gesù, IRCCS, Rome, Italy

**Keywords:** immunologic memory, hypogammaglobulinaemia, B cells, clinical immunology, pediatric nephrology, idiopathic nephrotic syndrome (INS)

## Abstract

Anti-CD20 therapy is effective in idiopathic nephrotic syndrome (INS). However, transient or sustained hypogammaglobulinemia predisposing to an increased risk of infectious diseases can follow treatment in some patients. We analyzed the long-term effects of anti-CD20 therapy on immunological memory in 27 frequently-relapsing/steroid-dependent INS pediatric patients after more than 4 years from the first and at least 2 years from the last anti-CD20 infusion. Twenty-one INS children, never treated with anti-CD20 and under an intense oral immunosuppression with prednisone, mycophenolate mofetil, and calcineurin inhibitors were also included as control group. Levels of circulating B-cell subpopulations, total serum immunoglobulins and IgG and memory B cells directed against hepatitis B virus (HBV) and tetanus were determined and correlated with clinical characteristics. Nine patients never relapsed after more than 2 years from the last anti-CD20 administration (5 after the first, 3 after the second, and 1 after the fifth infusion). At last follow-up, most patients showed a complete recovery and normalization of total (27/27), transitional (27/27), and mature-naïve B cells (25/27). However, a sustained and significant reduction of total memory (20/27) and switched memory (21/27) B cells was found in most patients. 11/27 patients showed hypogammaglobulinemia at last follow-up and, among these, four presented with a severe hypogammaglobulinemia (IgG < 160 mg/dl). In contrast, no patient in the control group developed a severe hypogammaglobulinemia. Age at the time of first anti-CD20 administration was positively associated with IgG levels at last follow-up (*p* = 0.008); accordingly, younger patients had an increased risk of hypogammaglobulinemia (*p* = 0.006). Furthermore, severe hypogammaglobulinemia and delayed switched memory B-cell reconstitution were more frequent in non-relapsing patients. Reduced IgG levels against HBV and tetanus were observed at baseline and further declined at last follow-up. Antigen-specific memory B-cells were induced by re-immunization, but specific IgG titers remained low. In conclusion, anti-CD20 therapy can be disease-modifying in some INS patients. However, a prolonged impairment of immunological memory occurs frequently, independently from the number of anti-CD20 infusions, particularly in younger patients. Re-immunization may be necessary in these patients.

## Introduction

Although anti-CD20 treatment is an off-label therapeutic approach for idiopathic nephrotic syndrome (INS), its efficacy in maintaining a prolonged remission in steroid-sensitive forms of the disease has been demonstrated by several randomized clinical trials (1–3). Anti-CD20 monoclonal antibodies transiently deplete all circulating B-cell subpopulations that express CD20, including transitional, naïve-mature and memory B cells. Conversely, plasma cells do not express CD20 and therefore are not affected by this treatment (4). Total B cells usually reappear after an average of 6 months after the last dose. However, a prolonged depletion of the memory B-cell compartment for more than 12 months after rituximab, a mouse-human chimeric anti-CD20 monoclonal antibody, has been reported in INS and other diseases, such as rheumatoid arthritis and systemic lupus erythematosus (5–7). In INS, a delayed reconstitution of the memory B-cell pool is associated to a prolonged remission despite tapering or discontinuation of concomitant immunosuppression (7). Although serum antibody levels are maintained by the function of CD20-negative long-lived plasma cells (4), several cases of hypogammaglobulinemia, occasionally associated with severe infections, have been recently described following rituximab treatment for different diseases (8, 9). A prospective study, aimed at monitoring changes in IgG levels in pediatric steroid-dependent INS treated with repeated rituximab infusions to maintain B-cell depletion for 18 months, reported an increased risk of prolonged hypogammaglobulinemia in patients with pre-existing low IgG levels (10). Reduced IgG levels were also observed after 2 years of a standardized protocol of four single rituximab infusions at 6-months interval in children with difficult-to-treat INS (11). In contrast, a retrospective short-term analysis of anti-CD20-related adverse events in a large cohort of multidrug-dependent INS children treated with rituximab or ofatumumab (a fully human anti-CD20 monoclonal antibody) showed normal total IgG levels and stability of anti-tetanus and anti-hepatitis B virus (HBV) IgG titers, albeit at 12-months (12).

To assess the long-term effects of anti-CD20 treatment in children with INS, we performed an observational study including patients with a minimum follow-up of 4 years from the first and of 2 years from the last anti-CD20 infusion. We measured the amount of total and specific B-cell subsets in the peripheral blood and the level of total and specific serum immunoglobulins. A long follow-up is necessary to assess reconstitution of the memory B-cell pool that occurs in response to antigen. As control group, we also included INS patients under an intense and prolonged oral immunosuppression with prednisone, mycophenolate mofetil (MMF) and/or calcineurin inhibitors (CNIs) but never treated with anti-CD20.

## Materials and Methods

### Study Patients

Twenty-seven frequently-relapsing (*n* = 2) or steroid-dependent nephrotic syndrome (*n* = 25) pediatric patients followed at the Ospedale Pediatrico Bambino Gesù, IRCCS (Rome, Italy) treated with anti-CD20 (rituximab and ofatumumab), with a minimum of 4 years follow-up after the first anti-CD20 infusion and of 2 years follow-up after the last infusion were enrolled in this observational study. Frequently-relapsing patients were defined as patients with 2 or more relapses observed over the last 6 months or 4 or more relapses observed within any 12-months period. Steroid-dependent NS was defined as frequently-relapsing NS with relapses occurred while still on steroids or within 2 weeks of discontinuing steroids (13). Relapse was defined as proteinuria of at least 3+ for at least three consecutive days by urine dipstick as previously described (13). Patients were treated with a single infusion of anti-CD20, followed by a second treatment at 7 days in case of non-complete depletion of total B cells, defined as CD19^+^ B cells > 10 cells/μl of the total peripheral blood lymphocytes assessed 2–7 days after the first infusion. All patients were treated with rituximab (administered at a dose of 375 mg/m^2^) at the first infusion. Within patients who received multiple infusions (≥2), only two patients were treated with ofatumumab (administered at a dose of 1,500 mg/1.73 m^2^) as last anti-CD20 administration. Anti-CD20 treatment was administered during corticosteroid-induced remission, and the infusion was repeated only in case of relapse for all patients except two—in which a rapid B-cell recovery was observed (1 and 3 months, respectively). Demographical and clinic characteristics, number of relapses, infectious disease occurrences, immunosuppressive treatment, such as prednisone, MMF and CNIs, and time to first relapse following each anti-CD20 infusion were also registered. After anti-CD20 treatment, the concomitant immunosuppressive therapy was gradually tapered or discontinued up to relapse, if it occurred.

Twenty-one frequently-relapsing (*n* = 4) or steroid-dependent (*n* = 17) INS patients never treated with anti-CD20, under a prolonged oral immunosuppression with prednisone, MMF and/or CNIs, and in complete remission, were also included as control group. These patients have been already included in a recent study evaluating the distribution of the different B-cell subpopulations in INS pediatric patients (14). Only patients >10 years old and in remission (comparable to anti-CD20-treated patients at last follow-up) were selected.

The amount of circulating B-cell subpopulations was monitored, and levels of total serum IgG, IgA, and IgM were determined before starting immunoglobulin replacement by intravenous (IVIg) or subcutaneous (SCIg) infusions in those patients who received it. Immunization against HBV, tetanus and measles/mumps/rubella (MMR) was also registered.

### Sample Procurement and Cell Isolation

Blood samples were obtained from included patients according to our institutional guidelines for informed consent, after approval from our local Ethics Committee and in compliance with the declaration of Helsinki. Blood sampling was performed at baseline (time of the first anti-CD20 infusion), after 2–7 days, after 1, 3, 6, 9, and 12 months, and at last follow-up. Peripheral blood mononuclear cells (PBMCs) were isolated by Ficoll-Paque Plus (Amersham Biosciences) density-gradient centrifugation.

### Flow Cytometry

To discriminate the different B-cell subpopulations, PBMCs were stained with fluorochrome-conjugated monoclonal Abs directed against CD19, CD24, CD27, CD38, IgD (BD Biosciences), and IgM (Jackson ImmunoResearch Laboratories) and then analyzed by multicolor flow cytometry (FACSCanto II; BD Biosciences). Subsets of gated CD19^+^ (total) B cells were identified based on the expression of surface markers as follows: transitional (CD38^high^CD24^high^) and mature-naïve (CD38^intermediate^CD24^low^), and expressed as absolute counts. Memory B cells were defined as CD19^+^CD27^+^ cells and memory subclasses were defined as IgM memory (IgM^+^IgD^intermediate^) or switched memory (IgM^−^IgD^−^) as previously described (7) and expressed as absolute counts. All analyses were performed with the FACSDiva software. Gated events (50,000) on living lymphocytes were analyzed for each sample. Age-related normal range was previously published (15).

### Immunoglobulin Levels

Total serum IgG, IgA, and IgM and anti-tetanus, anti-HBV, and anti-MMR IgG titers were measured as routine analysis in the diagnostic laboratory of Ospedale Pediatrico Bambino Gesù. Immunoglobulin deficiency that was observed at last follow-up but not at baseline was defined as a “*de-novo*” event.

### Evaluation of Tetanus and Hepatitis B-Specific B-Cell Responses

A Fluorospot assay was used for the quantification of antigen (Ag)-specific antibody (Ab) secreting cells. PBMCs were polyclonally activated *in vitro* in complete RPMI 1640 medium (Life Technologies, USA) supplemented with 0.5 μg/mL R848 (MabTech, Sweden) and 5 ng/mL interleukin-2 (MabTech, Sweden) and incubated for 3 days at 37°C. 96-well Fluorospot plates (MabTech, Sweden) were coated with 0.5 μg/well of Tetanus Toxoid non-adsorbed (NIBSC: 02/232) or Hepatitis B surface Antigen (NIBSC: 03/262) to evaluate the Ag-specific responses. Wells coated with keyhole limpet hemocyanin (0.5 μg per well) (Sigma-Aldrich, USA) were used as negative control, whereas wells coated with capture mAb MT91/145 (MabTech, Sweden), recognizing total IgG were used as positive control. After an overnight incubation at 4°C with the Ags, wells were blocked with 200 μl RPMI 1640 medium supplemented with 10% FBS (Life Technologies, USA) for 2 h at 37°C. Pre-stimulated PBMCs (2 ×10^5^ cells for Ag-coated wells or 5 ×10^4^ for controls) were added on the Fluorospot plates for 20 h at 37°C. After washing the plates, detection mAb anti-human IgG (MT78/145) (MabTech, Sweden) was added on the plates for 2 h at room temperature. The plates were developed using the fluorescence enhancer (MabTech, Sweden). Spots were counted using an AID FluoroSpot reader. Spots in the well with negative control were subtracted from the number of spots in the wells with Ag. Experiments were performed in triplicate and the number of spots were normalized for millions per cell. Competent response was defined as antigen-specific memory B cells ≥10 count/10^6^ cells, as determined by stimulation of total PBMCs isolated by nine healthy controls (five children and four adults).

### Statistical Analysis

Continuous data were expressed as mean and range if they passed normality test (Shapiro-Wilk test), or medians otherwise; categorical data are represented as numbers and percentages. Variables were compared by Student *t*-test, if normally distributed, or non-parametric Mann-Whitney *U*-test or Wilcoxon signed-rank test, as appropriate; Fisher's exact test was used for categorical variables. One way ANOVA was used for comparison of multiple groups and, if significant, pairwise comparisons were evaluated by the Bonferroni *post-hoc* analysis. The correlation of several parameters with the levels of serum IgG at baseline and at last follow-up, and with the rate of infections was analyzed by linear regression model. The association between age at time of first anti-CD20 infusion and risk of hypogammaglobulinemia at last follow-up was evaluated by logistic regression model. Relapse-free survival time was analyzed by Kaplan-Meier method and log-rank test for the whole cohort of patients after the first and the second anti-CD20 infusion. All *p*-values are two-sided and considered statistically significant with *p* < 0.05. Analyses were performed through the software GraphPad Prism 6 and SPSS 20.0.

## Results

### Patient Characteristics

Table 1 reports the characteristics of the different groups of INS patients. Anti-CD20-treated patients were analyzed at baseline and after a median time of 74 (range 48–118) months from the first treatment and 70 [range 26–113] months from the last treatment (last follow-up). The mean age was 12.9 (range 5.8–21.2) years at baseline and 19.1 (range 9.6–27.0) years at last follow-up. Twenty patients (74%) relapsed within 3 years (range 6–32 months) (Figure S1). Eleven patients were treated with ≥2 anti-CD20 infusions (details can be found in Table S1). In total, nine patients (33%) never relapsed after the last anti-CD20 administration: five patients after more than 5 years from one anti-CD20 infusion, three patients after more than 2 years from two anti-CD20 infusions (Figure S1), and one patient after 26 months from five infusions. Anti-CD20 treatment reduced the mean number of relapses/year from 3.4 (range 1–5) to 0.6 (range 0–2) at last follow-up (Table 1; *p* < 0.001). Furthermore, oral immunosuppression was significantly tapered or discontinued. During the 12 months before the first anti-CD20 treatment, all patients were on prednisone and at least one steroid-sparing agent (MMF and/or CNIs) (Table 1). At last follow-up 10/27 patients had discontinued all immunosuppressive therapy, 6/27 patients were on prednisone, 4/27 were on CNIs, 14/27 were on MMF, and 2/27 were on prednisone and two steroid-sparing agents (Table 1).

**Table 1:** Characteristics of patients.

**Parameter**	**Unit**	**Anti-CD20-treated baseline (*n* = 27)**	**Anti-CD20-treated last follow-up (*n* = 27)**	**Intense oral immunosuppression (*n* = 21)**
**Demographics**				
Age, mean [range]	Years	12.9 [5.8–21.2]	19.1 [9.6–27.0]^d^	16.5 [10.4–21.5]^**^
Male sex	N (%)	18 (67)	Same	14 (67)
**Disease characteristics**				
Age at disease onset, mean [range]	Years	5.1 [2.0–13.7]	Same	7.2 [1.7–18]
Steroid-dependent vs. frequently-relapsing nephrotic syndrome	Number	25 vs. 2	Same	17 vs. 4
Minimal change disease vs. focal segmental glomerulosclerosis^a^	Number	13 vs. 6	Same	8 vs. 5
**Relapses/year**	Number	3.4 [1–5]	0.6 [0–2]^***^	3.0 [2–5]^†††^
**Serum Albumin, mean [range]**	g/dl	4.4 [3.9–5.0]	4.6 [4.0–5.4]	4.3 [3.5–5.0]^†^
**U prot/creat ratio, mean [range]**	mg/mg	0.10 [0.03–0.2]	0.05 [0.02–0.15]^**^	0.10 [0.01–0.2]^†^
**Immunosoppressive drugs in the previous 12 months**	N (%)	27 (100)	17 (63)^***^	21 (100)^††^
Prednisone	N (%)	27 (100)	6 (22)^***^	20 (95)^†††^
Calcineurin inhibitors	N (%)	17 (63)	4 (15)^***^	8 (38)
Mycophenolate mofetil	N (%)	20 (74)	14 (52)	16 (76)
Calcineurin inhibitors or mycophenolate mofetil	N (%)	27 (100)	16 (59)^***^	19 (90)^†^
Calcineurin inhibitors and mycophenolate mofetil	N (%)	10 (37)	2 (7)^***^	5 (24)
**B cell subsets below the age-related normal range^b^**				
CD19 positive < 100 cells/μl	N (%)	6 (24)^e^	0 (0)^**^	4 (19)^†^
Transitional < 0 cells/μl	N (%)	0 (0)^e^	0 (0)	0 (0)
Mature < 60 cells/μl	N (%)	9 (36)^e^	2 (7)^*^	6 (29)
Memory < 30 cells/μl	N (%)	6 (24)^e^	20 (74)^***^	4 (19)^†††^
IgM memory < 10 cells/μl	N (%)	6 (24)^e^	12 (48)	4 (19)
Switched memory < 20 cells/μl	N (%)	8 (32)^e^	21 (78)^**^	7 (33)^††^
**Immunoglobulins below the normal range^c^**				
IgG < 600 mg/dl (at baseline) or 700 mg/dl (at last follow-up)	N (%)	13 (50)^f^	11 (41)	11 (65)^g^
IgG < 160 mg/dl (severe hypogammaglobulinemia)	N (%)	0 (0)^f^	4 (15)	0 (0)^g^
IgA < 70 mg/dl	N (%)	4 (15)^f^	7 (26)	3 (18)^g^
IgA < 10 mg/dl (severe IgA deficiency)	N (%)	0 (0)^f^	4 (15)	0 (0)^g^
IgM < 40 mg/dl	N (%)	3 (12)^f^	1 (4)	1 (6)^g^
**Impaired antigen-specific IgG titer^c^**				
Anti-HBV < 10 mIU/ml	N (%)	16 (62)^f^	20 (74)	10 (63)^h^
Anti-tetanus < 0.1 IU/ml	N (%)	7 (27)^f^	9 (33)	4 (27)^i^
Anti-tetanus < 0.6 IU/ml	N (%)	23 (88)^f^	24 (89)	12 (80)^i^
**Impaired antigen-specific memory B cells**				
Anti-HBV < 10 count/10^6^ cells	N (%)	–	10 (59)^g^	–
Anti-tetanus < 10 count/10^6^ cells	N (%)	–	11 (65)^g^	–

*Continuous data are expressed as mean and range, compared using unpaired t-test or one way ANOVA, and pairwise comparisons were evaluated by a Bonferroni post-hoc analysis*.

*Categorical values are indicated as absolute count and percentage, compared by a Fisher's exact test*.

a *Biopsy was not performed in eight patients*.

b *Range indicated in Piatosa et al. (15)*.

c *Range indicated in the diagnostic laboratory of our Institution. The age-related normal range for IgG is different between baseline and last follow-up due to a gap of ≥4 years between the two time-points*.

d *Per inclusion criteria, all patients had at least 4 years of follow-up*.

e *N = 25*.

f *N = 26*.

g *N = 17*.

h *N = 16*.

i *N = 15*.

* *p < 0.05 vs. Baseline*;

** *p < 0.01 vs. Baseline*;

*** *p < 0.001 vs. Baseline*.

† *p < 0.05 vs. Last Follow-up*;

†† *p < 0.01 vs. Last Follow-up*;

††† *p < 0.001 vs. Last Follow-up*.

In the control group (age range 10.4–21.5 years), 20/21 patients were on prednisone, 8/21 on CNIs, and 16/21 on MMF, at time of sampling, therefore a higher number of control patients was on prednisone compared to anti-CD20-treated patients at last follow-up, but MMF and CNIs treatments were comparable between the two groups (Table 1).

### B-Cell Subset Recovery

**Figure 1:**
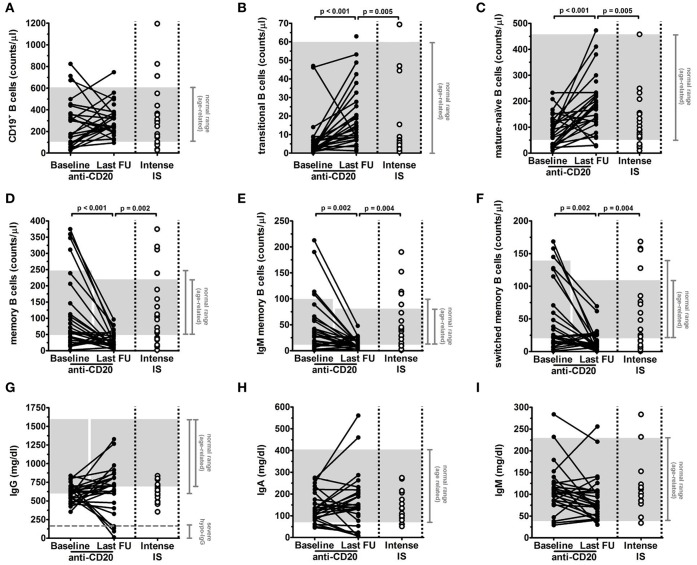
Long-term effects of anti-CD20 treatment on levels of circulating B-cell subsets and total serum immunoglobulins in children with idiopathic nephrotic syndrome (INS). Levels of B-cell subsets **(A–F)** and of immunoglobulins **(G–I)** were analyzed in INS pediatric patients (*n* = 27) before (Baseline) and after more than 4 years from the first and at least 2 years from the last anti-CD20 infusion (Last FU) and in INS patients under an intense oral immunosuppression (Intense IS, *n* = 21). **(A–F)** Gated total CD19^+^ B cells **(A)** were identified on the basis of the expression of surface markers as previously described (7); **(B)** transitional, **(C)** mature-naïve, **(D)** total memory, **(E)** IgM memory, and **(F)** switched memory B cells were expressed as absolute cell count per microliter of blood. **(G–I)** Levels of total **(G)** IgG, **(H)** IgA, and **(I)** IgM were expressed as mg/dl. Each plot represents a different patient. Gray areas represent the age-related normal range between the 5th and 95th percentiles indicated in Piatosa et al. (15) for the different B-cell subsets and by the diagnostic laboratory of our Institution for immunoglobulins. Levels of severe hypogammaglobulinemia (severe hypo-IgG) were identified by the dashed gray line in **(G)**. Since there is a gap of ≥4 years between baseline and last follow-up as per inclusion criteria, the age-related normal range can be different between the two time-points. Differences between groups were compared using the non-parametric paired Wilcoxon signed-rank test and unpaired Mann-Whitney U test, as appropriate.

**Figure 2:**
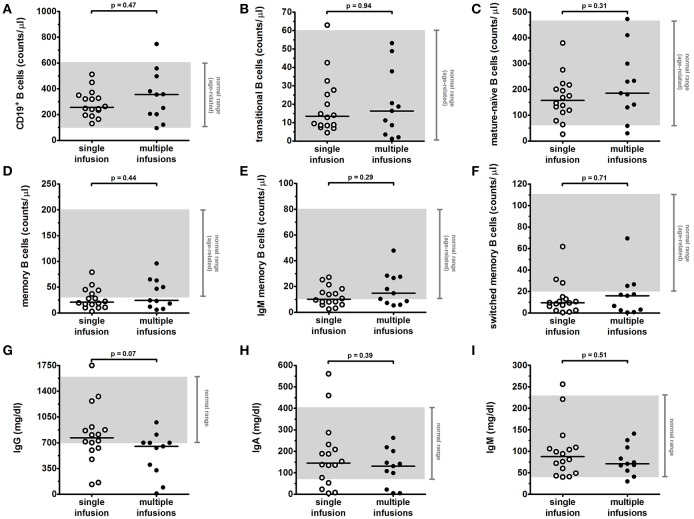
Long-term effects of single vs. multiple anti-CD20 infusions on levels of circulating B-cell subsets and total serum immunoglobulins in children with INS. Levels of B-cell subsets **(A–F)** and of immunoglobulins **(G–I)** were analyzed in INS pediatric patients as described in Figure 1 at last-follow-up following anti-CD20 treatment. Patients who received a single infusion (*n* = 16) were compared to patients who received multiple (≥2) infusions (*n* = 11) of anti-CD20. Each plot represents a different patient after single (with) or multiple (black) infusions. Gray areas represent the age-related normal range between the 5th and 95th percentiles indicated in Piatosa et al. (15) for the different B-cell subsets and by the diagnostic laboratory of our Institution for immunoglobulins. Differences between groups were compared using the non-parametric unpaired Mann-Whitney *U* test.

Absolute counts of B-cell subsets were determined as described in Material and Methods section. At baseline, total B-cell levels were above the normal range in 3/25 patients and below the normal range in 6/25 patients; mature B-cell levels were reduced in 9/25 patients and total memory, IgM memory and switched memory B-cell levels were reduced in 6/25, 6/25, and 8/25 patients, respectively (Table 1 and Figure 1). Total B-cells were depleted after anti-CD20 treatment and reappeared within 12 months in all patients (median time 6 months; Table S1). At last follow-up, total B-cells completely recovered (*p* = 0.36 comparing median levels between baseline and last-follow-up; Figure 1A) in all patients. However, the distribution of the different B-cell subsets was altered in treated patients. Specifically, we observed a significant increase of the median count of transitional (*p* < 0.001; Figure 1B) and mature-naïve B cells (*p* < 0.001; Figure 1C). Conversely, median levels of total memory B cells (*p* < 0.001; Figure 1D), IgM memory B cells (*p* = 0.002; Figure 1E), and switched memory B cells (*p* = 0.002; Figure 1F) were significantly decreased at last follow-up compared to baseline. Of note, no significant difference was observed comparing patients who received a single infusion to patients who received multiple infusions of anti-CD20 monoclonal antibody (Figures 2A–F; Table 2).

**Table 2:** Comparison between patients treated with single vs. multiple anti-CD20 infusions at last follow-up.

**Parameter**	**Unit**	**Single infusion (*n* = 16)**	**Multiple infusions (*n* = 11)**	***P*-value**
**B-cell subsets below the age-related normal range^a^**				
CD19 positive < 100 cells/μl	N (%)	0 (0)	0 (0)	1.00
Transitional < 0 cells/μl	N (%)	0 (0)	0 (0)	1.00
Mature < 60 cells/μl	N (%)	1 (6)	1 (9)	1.00
Memory < 30 cells/μl	N (%)	13 (81)	7 (64)	0.39
IgM memory < 10 cells/μl	N (%)	9 (56)	4 (36)	0.44
Switched memory < 20 cells/μl	N (%)	13 (81)	8 (73)	0.66
**Immunoglobulins below the normal range^b^**				
IgG < 700 mg/dl	N (%)	5 (31)	6 (54)	0.26
IgG < 160 mg/dl (severe hypogammaglobulinemia)	N (%)	2 (13)	2 (18)	1.00
IgA < 70 mg/dl	N (%)	4 (25)	3 (27)	1.00
IgA < 10 mg/dl (severe IgA deficiency)	N (%)	2 (13)	2 (18)	1.00
IgM < 40 mg/dl	N (%)	0 (0)	1 (0)	0.41
**Impaired antigen-specific IgG titer**^b^				
Anti-HBV < 10 mIU/ml	N (%)	11 (69)	9 (82)	0.66
Anti-tetanus < 0.1 IU/ml	N (%)	3 (19)	6 (54)	0.09
Anti-tetanus < 0.6 IU/ml	N (%)	15 (94)	9 (82)	0.55
**Impaired antigen-specific memory B cells**				
Anti-HBV < 10 count/10^6^ cells	N (%)	7 (58)^c^	3 (60)^d^	1.00
Anti-tetanus < 10 count/10^6^ cells	N (%)	8 (67)^c^	3 (60)^d^	1.00

*Data were compared by a Fisher's exact test*.

a *Normal range indicated in Piatosa et al. (15)*.

b *Range indicated in the diagnostic laboratory of our Institution*.

c *N = 12*.

d *N = 5*.

By comparison with control group, anti-CD20 treated patients at last follow-up had highest median levels of transitional and mature-naïve B cells (*p* = 0.005 for both populations), but a reduced proportion of the memory B-cell compartment (*p* = 0.002 for total memory B cells, *p* = 0.004 for IgM and switched memory B cells; Figure 1).

Comparable results were obtained by evaluating number of patients with values below the normal range of the different B-cell subsets (Table 1).

### Serum Immunoglobulin Levels

**Figure 3:**
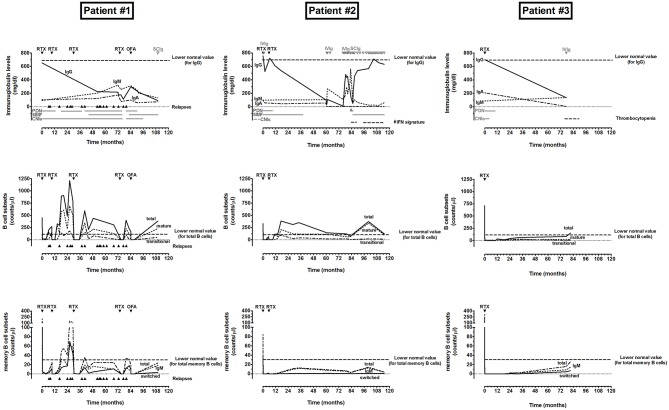
Monitoring of disease course, immunoglobulin and B-cell subset levels in INS patients who developed a severe *de-novo* hypogammaglobulinemia following anti-CD20 treatment. RTX, rituximab; OFA, ofatumumab; SCIg, subcutaneous immunoglobulin infusion; IVIg, intravenous immunoglobulin infusion; PDN, prednisone; MMF, mycophenolate mofetil; CNIs, calcinuerin inhibitors, IFN, interferon.

Serum immunoglobulin concentrations were analyzed at baseline and at last follow-up (Figures 1G,H and Table 1). When considering the entire cohort, no significant variation was observed between baseline and last follow-up for median levels of IgG (701 vs. 610 mg/dl at baseline; *p* = 0.19) and IgA (138 vs. 124 mg/dl at baseline; *p* = 0.53). A slight reduction was observed for IgM median levels (76 vs. 104 mg/dl at baseline; *p* = 0.05). At baseline, low IgG (<600 mg/dl), IgA (<70 mg/dl), and IgM (<40 mg/dl) levels were observed in 13/26, 4/26, and 3/26 patients, respectively (Table 1), whilst 2/26 patients had IgM levels above the normal range (Figure 1G). No patient presented with a severe hypogammaglobulinemia or IgA deficiency at this time-point (Table 1). 6/13 patients with low IgG at baseline (IgG < 600 mg/dl) had IgG levels below the normal range for age (IgG < 700 mg/dl) also at last follow-up, and one of them developed a severe hypogammaglobulinemia (IgG < 160 mg/dl). Furthermore, a *de-novo* hypogammaglobulinemia (IgG < 700 mg/dl) occurred in 5/13 patients who had normal IgG levels at baseline. This *de-novo* hypogammaglobulinemia was severe (IgG < 160 mg/dl) in three patients and prompted to IgG replacement. Details on disease course, immunoglobulin and B-cell subset levels for these three patients can be found below in the text and in Figure 3. Of note, the prevalence of hypogammaglobulinemia was similar between patients who received a single anti-CD20 infusion (5/16) and patients who received multiple (≥2) infusions (6/11) (*p* = 0.26; Table 2 and Figure 2G). In addition, a severe *de-novo* IgA deficiency (IgA < 10 mg/dl) occurred in four patients (two of them were those with severe hypogammaglobulinemia), independently of the number of anti-CD20 infusions, whilst only one patient had low IgM at last follow-up (Figures 1H,I, 2H,I). In contrast to anti-CD20 treated patients, no case in the control group of patients under intense oral immunosuppression had severe hypogammaglobulinemia or severe IgA deficiency, whereas 11/17 and 3/17 patients had low IgG (<700 mg/dl) or IgA (<70 mg/dl) levels, respectively, and 1/17 patients had low IgM (<40 mg/dl) (Figure 1; Table 1).

We also evaluated by linear regression model the contribution of several variables to serum IgG levels (Table 3). No significant correlation was observed with the intensity of immunosuppression both in terms of number of immunosuppressive drugs and combination regimens with the amount of the different B-cell subsets, both at baseline and at last follow-up (Table 3). Furthermore, prolonged maintenance or introduction of MMF and CNIs treatment during B-cell repopulation was not significantly correlated to levels of serum IgG at last follow-up (Table 3). At baseline, serum IgG levels inversely correlated with the number of relapses in the previous 12 months (*r* = −0.56, *p* = 0.004), but not with serum albumin (*r* = 0.08, *p* = 0.70) or urinary protein/creatinine ratio (*r* = 0.01, *p* = 0.96) at time of sampling. In contrast, total IgG levels at last follow-up were not associated with the frequency of relapses following anti-CD20 treatment (*r* = −0.15, *p* = 0.45), or with serum albumin (*r* = 0.15, *p* = 0.46) or urinary protein/creatinine ratio (*r* = −0.27, *p* = 0.18), but were positively correlated with the age at first anti-CD20 infusion (*r* = 0.50, *p* = 0.008). Of note, younger age at the time of first anti-CD20 infusion was significantly associated with an increased risk of hypogammaglobulinemia by logistic regression (odds ratio: 2.14/year, 95% confidence interval: 1.25–3.68; *p* = 0.006).

**Table 3:** Linear regression between several parameters, determined at baseline and at last follow-up, and levels of IgG at baseline, IgG at last follow-up and number of infections.

**Parameter**	**Unit**	**IgG at baseline**		**IgG at last follow-up**		**Number of infections**	
		**At baseline**	**At last follow-up**	**At baseline**	**At last follow-up**	**At baseline**	**At last follow-up**
Age	Years	0.20^a^	–	0.50^**^	0.35	−0.18	−0.13
Relapses/year	Number	−0.56^a^^,^^**^	–	−0.23	−0.15	0.04	0.20
Immunosuppressive drugs	Number	0.01^a^	–	−0.07	−0.10	0.02	0.09
Prednisone	Yes	–	–	–	−0.14	–	0.11
Calcineurin inhibitors	Yes	0.16^b^	–	−0.34	0.05	0.12	0.41^*^
Mycophenolate mofetil	Yes	−0.17^b^	–	0.30	−0.10	−0.12	−0.22
Immunosuppressive drugs during b-cell repopulation^d^							
Calcineurin inhibitors	Yes	–	–	–	0.15	–	0.14
Mycophenolate mofetil	Yes	–	–	–	0.33	–	−0.09
CD19 positive	Cells/μl	−0.24^c^	–	−0.23^b^	0.15	0.24^b^	−0.19
Transitional	Cells/μl	−0.29^c^	–	−0.01^b^	−0.15	0.23^b^	−0.05
Mature	Cells/μl	−0.12^c^	–	−0.22^b^	0.12	0.31^b^	−0.20
Memory	Cells/μl	−0.29^c^	–	−0.15^b^	0.15	0.12^b^	−0.06
IgM memory	Cells/μl	−0.35^c^	–	−0.04^b^	0.07	0.20^b^	−0.02
Switched memory	Cells/μl	−0.19^c^	–	−0.25^b^	0.22	0.08^b^	−0.19
IgG	mg/dl	–	–	0.17^a^	–	0.03^a^	−0.36
IgA	mg/dl	0.26^a^	–	−0.05^a^	0.35	0.06^a^	−0.15
IgM	mg/dl	0.35^a^	–	0.22^a^	0.14	−0.17^a^	0.04
Serum Albumin	g/dl	0.08^a^	–	0.14^a^	0.15	0.19^a^	−0.15
Urinary prot/creat ratio	mg/mg	0.01^a^	–	−0.04^a^	−0.27	−0.01^a^	0.19

a *N = 26*.

b *N = 25*.

c *N = 24*.

d *Maintenance of IS drugs for >12 months or introduction of IS treatment within 6 months after anti-CD20 administration*.

* *p < 0.05*;

** *p < 0.01*.

### Vaccine Competence

**Figure 4:**
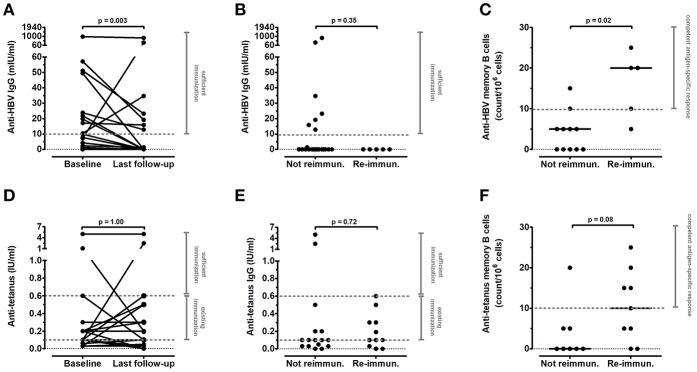
Long-term effects of anti-CD20 treatment on vaccine competence in children with INS. Antigen-specific IgG titers and memory B cells against **(A–C)** hepatitis B virus (HBV) and **(D–F)** tetanus were determined in INS pediatric patients before and after anti-CD20 treatment. **(A,D)** IgG titers compared between baseline and last follow-up (*n* = 27). **(B,E)** IgG titers at last follow-up comparing re-immunized (*n* = 5 against HBV and *n* = 11 against tetanus) vs. not re-immunized patients. **(C,F)** memory B cells evaluated by fluorospot analysis at last-follow-up (*n* = 17) comparing re-immunized (*n* = 5 against HBV and *n* = 9 against tetanus) vs. not re-immunized patients. Each plot represents a different patient. Protective levels identified by dashed gray lines were indicated in the diagnostic laboratory of our Institution. Differences between groups were compared using the non-parametric paired Wilcoxon signed-rank test or non-parametric unpaired Mann-Whitney U test, as appropriate.

The anti-tetanus and anti-HBV immunization was analyzed in all patients. At baseline, all patients had been vaccinated against HBV and tetanus as per national requirements. Reduced antigen-specific IgG titers were observed in a large number of anti-CD20-treated patients already at baseline (16/26 against HBV and 23/26 against tetanus; Table 1) and in the control group under intense oral immunosuppression (10/16 against HBV and 12/15 against tetanus; Table 1). Median anti-HBV IgG titers, but not anti-tetanus IgG titers, were significantly reduced at last follow-up compared to baseline (*p* = 0.003 and *p* = 1.00, respectively; Figures 4A,D). We also analyzed the number of antigen-specific memory B cells in 17 patients following anti-CD20 treatment. Only 5/27 patients (19%) were re-immunized against HBV after a mean time of 51 months (range 23–81 months) from the last anti-CD20 infusion, and 11/27 patients (41%) were re-immunized against tetanus after a mean time of 36 months (range 10–82 months). Total B-cell reappearance was verified in all of these patients when they were re-immunized. Re-immunization was not efficient in significantly increasing median levels of anti-HBV IgG (which were undetectable in 5/5 re-immunized patients; Figure 4B) or anti-tetanus IgG (which were below sufficient protection in 10/11 re-immunized patients; Figure 4E). However, a competent memory B-cell response (≥10 count/10^6^ cells) was observed in 4/5 patients re-immunized against HBV and 5/9 patients re-immunized against tetanus, respectively, compared with not re-immunized patients (*p* = 0.02 and *p* = 0.08 comparing median counts of anti-HBV and anti-tetanus memory B cells, respectively; Figures 4C,F). We have also analyzed MMR vaccine competence in four patients lacking protective IgG titers after anti-CD20 treatment. They were re-immunized 74 ± 7 months after the last anti-CD20 administration and protective IgG titers against MMR rose in 2/4 patients (50%).

### Adverse Events

Adverse events occurred in 18 patients (67%) (Table S1). In particular, infections occurred in 12 patients (44%) and moderate (IgG < 700 mg/dl) to severe (IgG < 160 mg/dl) hypogammaglobulinemia was observed in 7 (26%) and 4 (15%) patients at last follow-up, as reported above. Of note, a higher infection rate was associated with maintenance of calcineurin inhibitor treatment (*r* = 0.41, *p* = 0.03) but only slightly correlated with low serum IgG levels (*r* = −0.36, *p* = 0.06) at last follow-up (Table 3).

### Case Report of Occurred Severe Hypogammaglobulinemia

As reported above, a severe *de-novo* hypogammaglobulinemia (IgG < 160 mg/dl) developed in three patients, prompting to IgG replacement (Figure 3). All three patients experienced more than three relapses in the 12 months preceding the first anti-CD20 infusion with a steroid-dependent form of the disease and had normal IgG and IgA levels at baseline.

Patient number 1, a 6-years old girl with an initial steroid-resistant INS arisen at the age of 2 years that became steroid-dependent following treatment with cyclophosphamide and plasmapheresis, experienced 17 relapses during the follow-up (110 months), and was subjected to five different anti-CD20 administrations (four rituximab and one ofatumumab treatment). The last relapse occurred after 5 months from four rituximab infusions, despite introduction of MMF to support prednisone and CNIs immunosuppression between the third and the fourth administration. A fifth anti-CD20 infusion (with ofatumumab instead of rituximab due to early relapse after the fourth infusion) was administered 84 months from the first administration, and tacrolimus treatment was switched to cyclosporine treatment. The patient never relapsed after 26 months from ofatumumab administration and successful withdrawal of prednisone and cyclosporine (6 and 11 months after the fifth anti-CD20 administration) was completed. IgG levels, which were normal at baseline (650 mg/dl), were strongly reduced during the entire follow-up (<300 mg/dl), probably due to frequent relapsing episodes, but were further reduced following the last anti-CD20 infusion and prompted to IgG replacement started at last follow-up (IgG = 93 mg/dl), whereas levels of IgA and IgM were normal. Complete recovery of total CD19^+^ and memory B cells was observed after each cycle of anti-CD20 treatment. In contrast, switched memory cells, which reappeared after the first four cycles of treatment, were still very reduced at last follow-up.

Patient number 2, a 11 years-old boy with a steroid-dependent form of disease, was treated with prednisone and CNIs for several years, and MMF was introduced to interrupt CNIs 4 months before the first anti-CD20 infusion (with rituximab). A rapid B-cell recovery was observed (at 3 months) and a second anti-CD20 infusion (with rituximab) was administered 6 months after the previous administration. He never relapsed during the entire follow-up (118 months), and prednisone and MMF were discontinued following 9 and 38 months, respectively. IgG levels, normal at baseline (756 mg/dl), decreased rapidly after the first rituximab infusion and required a single IVIg administration (at 1 month) that completely restored their levels (722 mg/dl at 6 months). Following the second rituximab infusion, IgG levels precipitated, and a severe hypogammaglobulinemia (nadir IgG levels = 11 mg/dl) developed 5 years following the first rituximab, prompting to a prolonged IgG replacement (started with IVIg but switched to SCIg due to IVIg reactions) that is still necessary at the last follow-up. In parallel with development of hypogammaglobulinemia, IgA completely disappeared and never recovered, and an abnormal increase of IgM developed 18 months after onset of hypogammaglobulinemia, and persisted for 6 months. He experienced several infections (EBV, HHV6, pneumonia), lymphadenopathy and an increase of interferon (IFN) signature, which required immunosuppression with prednisone (discontinued after 2 months) and MMF (still present at the last follow-up). No causative genetic mutation leading to hyper-IgM or interferonopathy was found. A complete recovery of total CD19^+^ B cells was observed 6 months after the second rituximab infusion. In contrast, memory B cells were strongly reduced and a complete depletion of the switched memory B-cell subset was observed during the entire follow-up. *In vitro* stimulation of B cells failed to induce differentiation of IgG-secreting plasma cells.

Patient number 3, a 6 years-old girl with a steroid-dependent form of disease, was treated with prednisone and CNIs for several years before the first anti-CD20 infusion (with rituximab). Total B-cell reappearance was very slow (12 months), and no other anti-CD20 infusion was administered. The patient never relapsed during the entire follow-up (90 months), and CNIs and prednisone were discontinued following 4 and 10 months, respectively. IgG and IgA levels were normal at baseline (700 mg/dl and 206 mg/dl), but resulted strongly reduced at last follow-up (134 and <5 mg/dl, respectively). She experienced a mild thrombocytopenia (nadir value 103 ×10^3^ platelets/μl) following 77 months (still present at the last follow-up) and a pneumonia infection, which prompted to IVIg administration at last follow-up. A normalization of total CD19^+^ B cells was observed only at last follow-up, and the reappearance of total memory B cells and in particular of switched memory B cells was strongly delayed.

All three patients had undetectable anti-tetanus and anti-HBV IgG titers at last follow-up, whereas all of them were immunized against HBV and tetanus during the first year of life and two of them were re-immunized against tetanus after 12 and 80 months following the last anti-CD20 infusion. Patient number 3 was also evaluated for her amount of antigen-specific memory B cells, which resulted below the protective levels against tetanus but not against HBV.

### Clinical Response and Relation to IgG and Switched Memory B-Cell Levels

**Figure 5:**
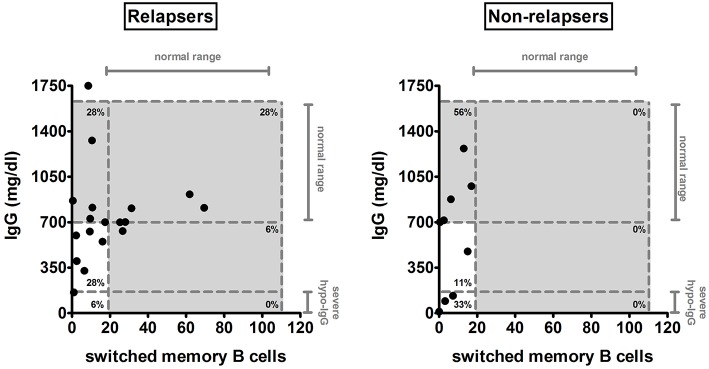
IgG and switched memory B-cell levels at last follow-up in relapsing and non-relapsing patients after anti-CD20 treatment. The amount of serum IgG and of switched memory B cells was compared between relapsing (*n* = 18) and non-relapsing (*n* = 9) anti-CD20-treated patients at last follow-up. Each plot represents a different patient. Gray areas and dashed lines represent the age-related normal range between the 5th and 95th percentiles indicated in Piatosa et al. (15) for the different B-cell subsets and by the diagnostic laboratory of our Institution for immunoglobulins. Levels of severe hypogammaglobulinemia (severe hypo-IgG) are also indicated.

We also evaluated whether a prolonged response to anti-CD20 treatment could be associated to the time of switched memory B-cell recovery and/or occurrence of hypogammaglobulinemia (Figure 5). At last follow-up, none of the nine non-relapsers after the last anti-CD20 infusion had normal levels of switched memory B cells, in contrast to 6/18 relapsing patients (*p* = 0.07). Furthermore, 3/9 non-relapsers developed a severe hypogammaglobulinemia compared to 1/18 relapsing patients (*p* = 0.09). Of note, this relapsing patient with severe hypogammaglobulinemia had only one relapse, after which maintained a prolonged remission for more than 2 years, despite the tapering of concomitant immunosuppression.

## Discussion

In the current study, we characterized the long-term immunological effects of anti-CD20 treatment with rituximab and ofatumumab in children with steroid-sensitive INS. As far as clinical response is concerned, 9/27 patients never relapsed after more than 2 years from the last anti-CD20 infusion despite having tapered or discontinued concomitant immunosuppression. This finding confirms that anti-CD20 treatment can be disease-modifying in some INS pediatric patients [reviewed in (3)]. We also evaluated the effects of anti-CD20-induced B-cell depletion on long-term immunological memory. The entire circulating CD20^+^ B-cells, including transitional, mature-naïve, and memory subsets that express CD20 molecules on their cell surface can be efficiently depleted by anti-CD20 monoclonal antibodies. Short-lived plasma cells, which lack CD20 expression, rapidly wane due to their limited lifespan. Thus, anti-CD20 treatment transiently depletes the entire peripheral B-cell lineage. Following anti-CD20 clearance, replenishment initiates from bone marrow precursors (16). Peripheral B-cell reappearance usually requires 6 months in INS pediatric patients, and a complete recovery of total B-cell numbers can be observed 12 months after anti-CD20 infusion (7, 12). We have previously described that in children with INS, 1 year following anti-CD20 treatment, peripheral B cells are mainly constituted by transitional and mature-naïve B cells, whilst the memory B-cell compartment is still significantly reduced (7). In the current study, we prolonged follow-up to >2 years (median follow-up 74 months) and found a subsequent normalization of total, transitional and mature-naïve B cells, but a sustained and profound reduction of memory B cells, in particular of the switched memory B-cell population, in the vast majority of patients at the last follow-up. To exclude a role of intense oral immunosuppression in affecting memory B-cell levels independently of anti-CD20 treatment, we also characterized the distribution of the different B-cell subsets in a group of INS patients never treated with anti-CD20 but under an intense oral immunosuppression with prednisone and one or two steroid-sparing agents (MMF and/or CNIs). Levels of transitional and mature-naïve B cells resulted significantly reduced in this control group, whereas they showed significantly higher levels of memory B cells compared to the group of anti-CD20-treated patients at last follow-up. Similar results were previously reported in renal transplanted patients treated with steroids plus MMF and CNIs (17). Our results show that an intense oral immunosuppression mainly affects the amount of transitional and mature-naïve B cells, whilst memory B-cell levels were only partially affected by this treatment. In contrast, anti-CD20 therapy efficiently depletes all the circulating B-cell subsets.

We then analyzed serum immunoglobulin levels before and after anti-CD20 treatment. Previous studies in INS patients described a marked reduction of IgG levels and an increased IgM concentration in relapse, which can persist for several months also in remission (18, 19). Anti-CD20 treatment can induce a prolonged reduction of serum IgM in several autoimmune diseases (20), including adult systemic lupus erythematosus patients (21). In our INS population, median IgM concentrations were only slightly reduced at last follow-up, suggesting disease- and/or age-specific differences in children with INS. Of note, an intense oral immunosuppression with prednisone plus MMF and/or CNIs did not reduce IgM levels in the control intense oral immunosuppression group.

In contrast to IgM concentration, median levels of serum IgG did not differ between baseline and last follow-up following anti-CD20 treatment. However, we observed hypogammaglobulinemia in 11/27 patients at last follow-up, which was severe in four of them. Interestingly, five of these patients received only a single anti-CD20 infusion. The occurrence of hypogammaglobulinemia following multiple infusions of anti-CD20 has been already described in INS and in other diseases, sometimes associated with the presence of low IgG levels before anti-CD20 administration (8–10). In our patients, concomitant immunosuppression does not explain the occurrence of severe hypogammaglobulinemia, because this symptom was never observed in patients under an intense oral immunosuppression but never treated with anti-CD20. Accordingly, no significant correlation between serum IgG concentrations and immunosuppressive treatment at baseline, during B-cell repopulation, or at last follow-up was found in anti-CD20-treated patients, suggesting that an intense oral immunosuppression can only partially contribute to serum IgG reduction. In contrast, we observed that reduced serum IgG levels at baseline were associated with a high frequency of relapses in the previous 12 months, but not with serum albumin or urinary protein/creatinine ratio at time of sampling. Our results confirm that an intense disease activity can affect basal levels of serum IgG, which require more time than proteinuria or serum albumin to reach normal values after remission occurrence (18, 19). Of note, reduced IgG levels at last follow-up did not significantly correlate with the frequency of relapses following anti-CD20 administration or with serum albumin or proteinuria. On the contrary, patients who maintained a prolonged remission after anti-CD20 treatment were more prone to develop severe hypogammaglobulinemia. In parallel, a tendency to a further delay of the switched memory B-cell reconstitution was observed in non-relapsing patients, as previously described in INS and in other autoimmune diseases (5–7). Our observation suggests that intense and prolonged switched memory B-cell depletion can lead both to prolonged disease remission and to persistent severe hypogammaglobulinemia in some individuals. This has been described also in other disease following rituximab treatment [as reviewed in (9)], and poses a quandary to the pediatric nephrologist in terms of weighing relative risks and benefits of therapy with anti-CD20 antibodies for difficult forms of INS. Further studies identifying genetic markers of predisposition and therefore allowing identification of these patients before anti-CD20 administration would be therefore useful. However, the delayed recovery of the switched memory B cells was not *per se* sufficient to predict the development of hypogammaglobulinemia, since this B-cell subpopulation was still reduced in 21/27 patients at last follow-up, while only 11/27 patients presented hypogammaglobulinemia. Rather, a younger age at the time of first anti-CD20 administration was significantly predictive of low IgG levels at last follow-up. A possible explanation for this finding could reside in B-cell ontogeny. During infancy, the peripheral B-cell pool is constituted predominantly by transitional B cells, which migrate from the bone marrow to the periphery, where they acquire a naïve-mature phenotype. Here they encounter antigens that induce B-cell activation and differentiation into antibody-secreting plasma cells or memory B cells, which can in turn produce IgM or undergo isotype switching to IgG-, IgA-, or IgE-secreting B cells. Circulating plasma cells generally die after few days, but some long-lived plasma cells return to the bone marrow or migrate into other secondary lymphoid organs and are responsible for the maintenance of serum immunoglobulin levels in healthy conditions, independently of further antigen exposure (4, 22). Populating these secondary “niches” generally takes several years. A recent study evaluating the age-related levels of bone-marrow resident lymphoid cells shows that the amount of bone marrow plasma cells is very low for the entire childhood (23). Accordingly, B-cell depletion may affect the physiological formation of long-lived plasma cells more in children than in adults (24, 25). Furthermore, we have recently described an intrinsic B-cell dysregulation in INS pediatric patients at disease onset, independently from any treatment (14), which could contribute to an altered plasma-cell repertoire in the bone marrow and/or in secondary lymphoid organs of these children. This aspect deserves further investigations. Of note, IgA deficiency also occurred in four patients (two of them were those with severe hypogammaglobulinemia), further suggesting a defect in isotype-switched immunoglobulins unmasked by anti-CD20 treatment in some INS patients, as already hypothesized (18). Although in our cohort the patient with the most severe hypogammaglobulinemia did experience more severe infections, low IgG levels at last follow-up were only slightly correlated with an increased rate of infection, in accordance with previous reports on several autoimmune diseases treated with anti-CD20 monoclonal antibodies [reviewed in (9)]. In contrast, the rate of infections was more significantly associated with maintenance of calcineurin inhibitors. These results suggest that the risk of infection following anti-CD20 treatment can depend also on the concomitant immunosuppressive treatment, as previously reported (8). In this regard, treatment with steroids, MMF and/or CNIs can also impair vaccine-induced immunization against HBV and tetanus in INS (26, 27) and in other diseases (28). Accordingly, we observed an impaired antigen-specific vaccine response against HBV in 62% of patients and against tetanus in 88% of patients at baseline, and in 63 and 80% of patients under an intense oral immunosuppression never treated with anti-CD20, confirming recent findings described in multidrug-dependent INS children (12). Median levels of anti-HBV IgG were further significantly reduced following anti-CD20 therapy, and 5/5 patients re-immunized against HBV and 10/11 patients re-immunized against tetanus showed unprotective IgG titers at last follow-up. However, re-immunization induced a competent antigen-specific memory B-cell response in most patients, which was lacking in non-re-immunized patients, because all B cells, including memory B cells, had been depleted by anti-CD20 treatment. Antigen-specific IgG levels remained low in vaccinated INS patients suggesting either a relatively impairment of the germinal center reaction or a delayed reconstitution of the long-lived plasma cell niche. Although antigen-specific IgG titers are used as bio-marker after immunization, vaccine-specific memory B cells are indispensable to generate antibodies when they are needed thus exerting an effective protection (29, 30). Our data also suggest that re-immunization following anti-CD20 treatment is effective and should be considered for all patients who could have lost their vaccine competence despite total B-cell reconstitution. Furthermore, these results confirm that treatment with the standard therapy based on immunosuppressive agents that affect B- and T-cell function (31) can reduce vaccine competence in INS pediatric patients and suggest that anti-CD20 treatment can induce an additional impairment of vaccine response. This further impairment may be due to two concurrent factors: a reduction in levels of switched memory B cells, and a parallel reduction in the frequency of CD4^+^ T follicular helper cells, responsible for memory B-cell differentiation into antibody-secreting plasma cells, which has been described following anti-CD20 treatment in INS patients (32).

The main limitation of the current study is that anti-CD20 treatment was not standardized. Furthermore, due to the relatively low number of patients, we cannot subdivide patients according to the number of infusions. However, we observed a prolonged immunological impairment even after a single anti-CD20 infusion. Moreover, our stringent patient selection (follow-up of at least 2 years after the last anti-CD20 infusion) and evaluation of antigen-specific B-cell subsets and immunoglobulins, sheds light on the real impact that anti-CD20 treatment can have on immunological memory in children with INS.

In conclusion, our study highlights the importance of prolonged immunological monitoring of patients treated with anti-CD20 antibodies for INS, even after a single infusion and even if they had normal levels of B cells and total IgG at baseline. A reduced vaccine competence can indeed occur and a severe hypogammaglobulinemia can emerge after several years in patients with a prolonged remission, especially if they received the first infusion at an early age. Further studies are necessary to evaluate the clinical impact of hypogammaglobulinemia in patients with INS in prolonged remission.

## Data Availability

The datasets generated for this study are available on request to the corresponding author.

## Ethics Statement

This study was carried out according to Ospedale Pediatrico Bambino Gesù (OPBG) guidelines for informed consent, after approval from OPBG Ethics Committee and in compliance with the Declaration of Helsinki.

## Author Contributions

MC, MV, FE, RC, PP, LM, and AG: research idea and study design. MC, JS, SR, AL, and CC: data acquisition. MC, MV, RC, PP, LM, AG, NC, OP, and AO: data analysis/interpretation. MC and FE: statistical analysis. MV and FE: supervision or mentorship. All authors contributed to manuscript revision, read, and approved the submitted version.

### Conflict of Interest Statement

The authors declare that the research was conducted in the absence of any commercial or financial relationships that could be construed as a potential conflict of interest.
