# Supplementary material for: Prolonged Impairment of Immunological Memory After Anti-CD20 Treatment in Pediatric Idiopathic Nephrotic Syndrome
Source: Front Immunol. 2019 Jul 16;10:1653. doi: 10.3389/fimmu.2019.01653 (PMC6646679; doi:10.3389/fimmu.2019.01653)
Supplement: Supplementary file 1 [file Table_1.docx]

**Table S1. Anti-CD20 treatment and treatment-related adverse events.**

| **Parameter** | **Unit** | **Last Follow-up**  **(n = 27)** |
| --- | --- | --- |
| **Treatment** |  |  |
| One infusion^a^ | N (%) | 27 (100) |
| Multiple infusions^b^ | N (%) | 11 (41) |
| **B-cell depletion** | N (%) | 27 (100) |
| **B-cell reappearance** | N (%) | 27 (100) |
| < 1 month | N (%) | 1 (4) |
| < 3 months | N (%) | 7 (26) |
| < 6 months | N (%) | 20 (74) |
| < 9 months | N (%) | 26 (96) |
| < 12 months | N (%) | 27 (100) |
| **Serious adverse events** | N (%) | 18 (67) |
| Infections (pneumonia, RTI, EBV, HZV, HHV6, encephalitis, otitis) | N (%) | 12 (44) |
| Lymphatic disorders (lymphadenopathy, leukopenia) | N (%) | 2 (7) |
| Thrombocytopenia | N (%) | 1 (4) |
| Allergic episodes | N (%) | 2 (7)^c^ |
| Moderate hypogammaglobulinemia (IgG<700 mg/dl) | N (%) | 7 (26) |
| Severe hypogammaglobulinemia (IgG<160 mg/dl) | N (%) | 4 (15) |
| Other (H Pylorii gastritis, osteopenia, severe obesity, irritable colon, fibroadenoma, axillary abscess) | N (%) | 5 (19) |

^a^ All patients were treated with rituximab at the first infusion. ^b^ Ten patients received two infusions and 1 patient received 5 infusions; two patients were treated with ofatumumab as last anti-CD20 infusion (one for occurring allergic episode during the second rituximab infusion and one for early relapse following the 4^th^ rituximab infusion). ^c^ One patient experienced rash one week later and one patients experienced rash and dyspnea during the second infusion with rituximab. RTI, respiratory tract infection; EBV, Epstein Barr virus; HZV, herpes zoster virus; HHV6, human herpesvirus 6.
