# Supplementary figures and images for: Prolonged Impairment of Immunological Memory After Anti-CD20 Treatment in Pediatric Idiopathic Nephrotic Syndrome
Source: Front Immunol. 2019 Jul 16;10:1653. doi: 10.3389/fimmu.2019.01653 (PMC6646679; doi:10.3389/fimmu.2019.01653)

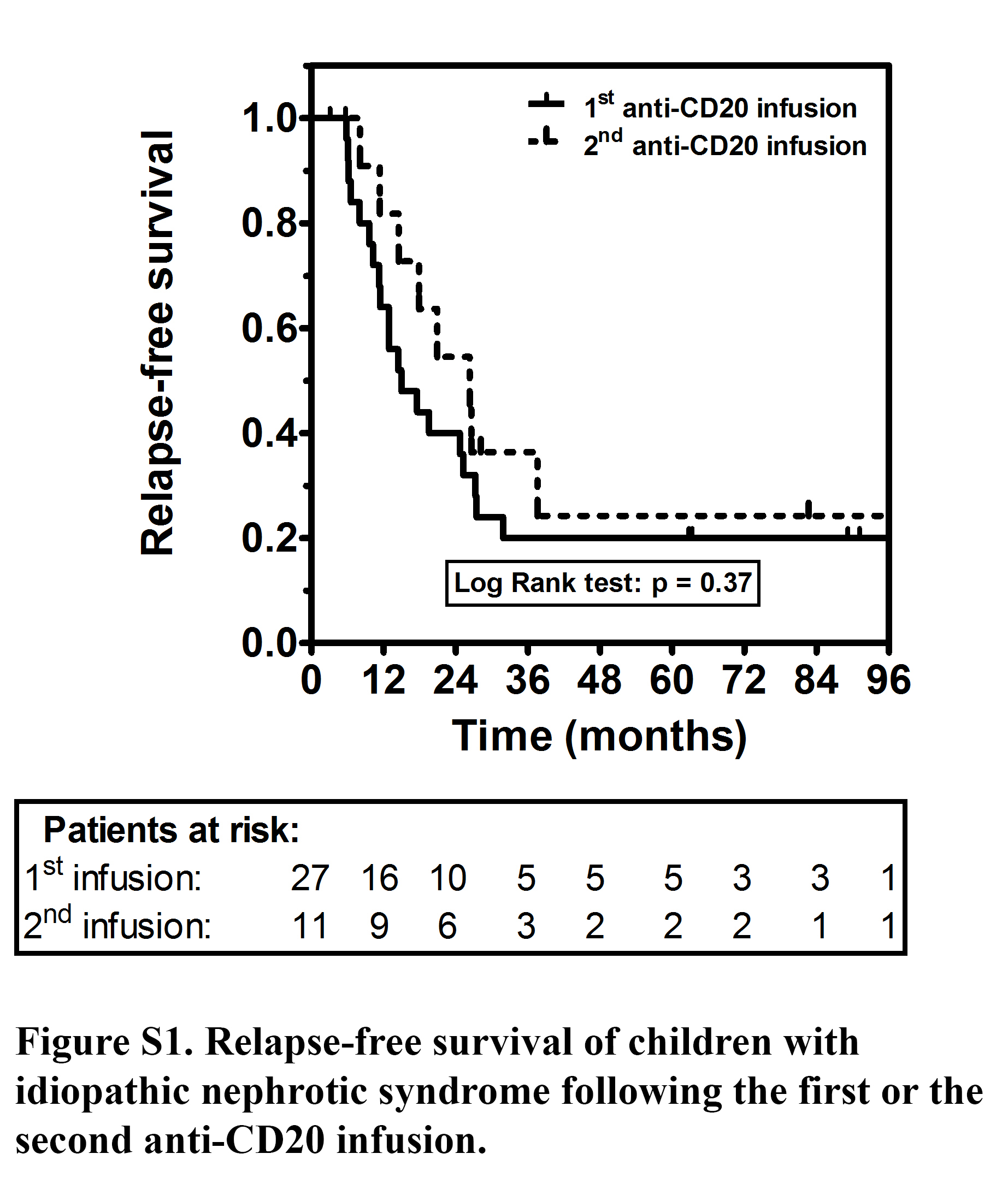

Supplement: Supplementary file 2 [file Image_1.JPEG]
